# Circadian rhythms in the three-dimensional genome: implications of chromatin interactions for cyclic transcription

**DOI:** 10.1186/s13148-019-0677-2

**Published:** 2019-05-15

**Authors:** Ignacio Pacheco-Bernal, Fernando Becerril-Pérez, Lorena Aguilar-Arnal

**Affiliations:** Instituto de Investigaciones Biomédicas, Departamento de Biología Celular y Fisiología, Universidad Nacional Autónoma de México, Mexico City, Mexico

**Keywords:** Environmental epigenetics, Circadian clocks, Chromatin, Genome topology, Transcription

## Abstract

Circadian rhythms orchestrate crucial physiological functions and behavioral aspects around a day in almost all living forms. The circadian clock is a time tracking system that permits organisms to predict and anticipate periodic environmental fluctuations. The circadian system is hierarchically organized, and a master pacemaker located in the brain synchronizes subsidiary clocks in the rest of the organism. Adequate synchrony between central and peripheral clocks ensures fitness and potentiates a healthy state. Conversely, disruption of circadian rhythmicity is associated with metabolic diseases, psychiatric disorders, or cancer, amongst other pathologies. Remarkably, the molecular machinery directing circadian rhythms consists of an intricate network of feedback loops in transcription and translation which impose 24-h cycles in gene expression across all tissues. Interestingly, the molecular clock collaborates with multitude of epigenetic remodelers to fine tune transcriptional rhythms in a tissue-specific manner. Very exciting research demonstrate that three-dimensional properties of the genome have a regulatory role on circadian transcriptional rhythmicity, from bacteria to mammals. Unexpectedly, highly dynamic long-range chromatin interactions have been revealed during the circadian cycle in mammalian cells, where thousands of regulatory elements physically interact with promoter regions every 24 h. Molecular mechanisms directing circadian dynamics on chromatin folding are emerging, and the coordinated action between the core clock and epigenetic remodelers appears to be essential for these movements. These evidences reveal a critical epigenetic regulatory layer for circadian rhythms and pave the way to uncover molecular mechanisms triggering pathological states associated to circadian misalignment.

## Background

Circadian rhythms are apparent in most living organisms, as persistent 24-h cycles in physiology, behavior, or even metabolism. They have evolved to adapt and anticipate daily environmental fluctuations, temporally segregating biological functions to specific hours along the day. For example, in humans, sleep/wake cycles are under circadian control, and they fluctuate in coherence with feeding/fasting periods. As a result, metabolic fluxes show rhythmic regulation in most tissues, allowing the organisms to efficiently couple food intake with adequate energy use [1]. Moreover, cognitive functions, such as learning or memory formation, are clock controlled, and circadian synaptic plasticity has been evidenced [2]. Hence, circadian control of physiology has broad implications, which manifest when the circadian clock is disrupted, leading to disease. For example, genetic or pharmacological perturbations of circadian rhythms can lead to obesity and type 2 diabetes and, remarkably, disruption of light/dark cycles or arbitrary feeding schedules are strongly associated with metabolic disorders [3]. Indeed, other pathological conditions including cardiovascular diseases, inflammation, cancer, or psychiatric disorders are also related to circadian misalignment [4]. Indeed, uncovering the rules governing circadian rhythms can pave the way to prevent and treat complex diseases prevalent in modern societies.

Classic and novel genetic and molecular biology approaches shed light on the gears of circadian rhythms at the cellular level [5]. Remarkably, the molecular components of the circadian clock are a combination of transcriptional activators and repressors coordinately acting at thousands of sites in the chromatin fiber and ultimately driving a highly specific program of gene expression around the day [5, 6]. Within each tissue, distinct genetic networks are controlled by the molecular clock, through synchronized action with tissue-specific transcription factors and epigenetic remodelers [7–9]. Complex regulatory networks intertwine to modulate the circadian clock, providing means to adapt the circadian program of gene expression to environmental signaling. In this scenario, chromatin dynamics plays a pivotal role on clock-controlled transcription, and it is conceptually apparent that organizational principles of the genome within the nuclear space contribute to rhythmic transcription, as it has been demonstrated in latest research. In this review, we will discuss findings uncovering the implications of epigenetic regulation genome folding on circadian clock function, with special emphasis on evidences relating three-dimensional properties of chromatin to cyclic transcription.

## The mammalian circadian clock

In mammals, the circadian clock is a time tracking system that allows the organism to anticipate and adapt to environmental changes. This system is organized as a hierarchy of oscillators, coordinated by a central pacemaker located in suprachiasmatic nucleus (SCN) of the hypothalamus. The SCN is composed of about 20,000 neurons with unique network properties that confer the entire nucleus a remarkable synchrony in its electrical properties, showing autonomous circadian cycles in the action-potential firing from these pacemaker neurons [10]. Remarkably, the SCN is enough to drive circadian behavior, and participates in the control of circadian physiology. Although it generates endogenous and self-sustained rhythms, the SCN must receive external cues to daily synchronize with the environment and keep the correct time. Interestingly, the SCN receives light information directly form a subset of retinal ganglion cells expressing the specific photopigment melanopsin, through the retinohypothalamic tract [11]. Blue light is therefore the strongest time giver in most of mammalian species, as it entrains the central pacemaker to keep it on time. However, subsidiary clocks in the rest of the body, which are not sensitive to photic entrainment, must be also synchronized. This is partially achieved by signaling from the SCN through the coordinated action of humoral, hormonal, and neural inputs into peripheral clocks, located at other areas of the brain and distinct tissues [11]. Additionally, certain environmental cues, such as food intake, temperature, exercise, and drug exposure, can synchronize peripheral clocks to reinforce the alignment between them or, on the contrary, disturb this synchrony when exposure is unfavorable, a case that can lead to disease [12]. Some of the pathologies driven by circadian misalignment are cardiovascular diseases, obesity and metabolic syndrome, mental disorders, or even cancer [12].

**Fig. 1:**
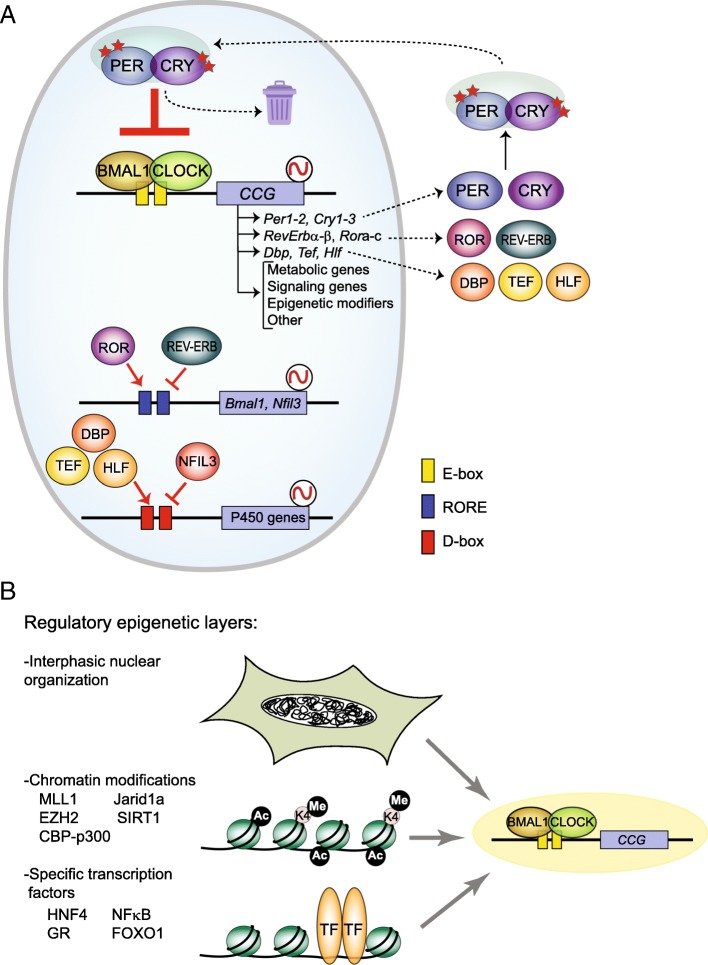
The mammalian circadian clock. **a** Schematic representation of the molecular clock. The transcriptional activators CLOCK:BMAL1 rhythmically bind to E-boxes (yellow rectangles) and activate expression of clock-controlled genes (CCG). Amongst these CCG, the circadian repressors *Cry1-2* and *Per1-3* are transcribed. Upon translation, Period complexes are ensembled in the cytoplasm, and distinct posttranslational modifications control its activity (red stars). Nuclear translocated Period complexes sequester CLOCK:BMAL1 transactivator heterodimers, hereby interrupting transcription of CCG. Proteasomal degradation fine tunes clearance of repressors from the nucleus. Reinforcing loops include the interplay between REV-ERB and ROR proteins at ROR elements (blue rectangles) in the promoters of many genes, including that of *Bmal1*. Additionally, certain transcription factors are clock controlled, as exemplified by the PAR-bZip family including the activators DBP, TEF, and HLF and the repressor NFIL3. Through binding to D-boxes in the genome (red rectangles), these can impose rhythmicity to specific genetic programs, such as demonstrated for components of the cytochrome P450 system in the mouse liver [106]. **b** Distinct epigenetic regulatory layers coordinate circadian transcriptional output. Tissue-specific transcription factors, nuclear receptors, or master regulators of intracellular signaling can determine a portion of the circadian transcriptome in response to environmental cues. Additionally, epigenetic modifications including DNA methylation or histone marks are highly dynamic and shape circadian transcription. Indeed, the global nuclear architecture has been demonstrated to coordinate transitions between transcriptionally active and repressive states during the circadian cycle

At the cellular level, a circadian molecular machinery coordinates rhythmicity by autoregulatory feedback loops in transcription and translation. Interestingly, the core components of this molecular gear are transcription factors which timely regulate their own synthesis and degradation in 24-h cycles. In mammals, the positive limb of the loop is driven by two b-HLH (b-helix-loop-helix) PER–ARNT–SIM (bHLH–PAS) domain proteins, CLOCK (Circadian Locomotor Output Cycles Kaput) and BMAL1 (Brain and Muscle ARNT-Like Protein-1), which heterodimerize and bind to the consensus motif e-box in the genome (Fig. 1a). CLOCK:BMAL1 recruitment to chromatin triggers nucleosome remodeling and primes genes for PolII-mediated transcriptional activation [13, 14]. Some of the genes activated by CLOCK:BMAL1 heterodimers are *Period* (*Per1-3*) and *Cryptochromes* (*Cry1-2*) genes, which when translated, dimerize together and with members of the casein kinase 1 family (CK1α, CK1ε) and conform the Period repressive complexes [15, 16]. Notably, Period complexes are subjected to multiple posttranslational modifications, such as phosphorylation, ubiquitylation, or acetylation, which modulate its activity [16, 17]. Period complexes translocate into the nucleus to displace CLOCK:BMAL1 from chromatin, hence transcriptionally silencing *Per* and *Cry* genes (Fig. 1a). Remarkably, Period complexes have very tightly controlled half-lives through specific E2-Ubiquitin ligases, which target them for proteasomal degradation. Clearance of period complexes releases CLOCK:BMAL1 activators which subsequently can reenter another cycle of transcriptional activation. Through this mechanism, a significant set of genes are expressed in 24-h cycles, corresponding to the length of the transcriptional-translational feedback loop [18].

Additional to the core clock TTFL, several regulatory loops interlock. For example, expression of the nuclear receptors (NRs) *Nr1d1 and Nr1d2* (also known as *REV-ERBα and REV-ERBβ*) is activated by CLOCK:BMAL1 (Fig. 1a). These NRs are transcriptional repressors that bind to the RevDR2 and retinoic acid-related orphan receptor (ROR)-binding elements (ROREs), which are also recognized by the ROR family of NRs, RORα, RORβ, and RORγ. This molecular crosstalk controls rhythmic *Bmal1* and *Cry1* expression, evidencing a specific circadian cistrome coordinating cyclic transcription [19] (Fig. 1a). Additional motifs involved in rhythmic gene expression are D-box elements, which are recognized by the PAR-bZip (proline and acidic amino acid-rich basic leucine zipper) activating transcription factors DBP (D-box binding protein), TEF (thyrotroph embryonic factor) and HLF (hepatic leukemia factor), and the repressor NFIL3 (nuclear factor, interleukin-3 regulated; also known as E4BP4). Remarkably, expression of PAR-bZip activators is driven by the core clock TTFL through binding to E-boxes in their promoters, while that of the repressor NFIL3 is preferably controlled by ROREs (Fig. 1a). This allows for the temporally segregated expression of activators and repressors, which in turn impose rhythmicity to subsidiary genes.

In this scenario, tissue-specific oscillations for distinct transcripts represent a challenge for fully understanding circadian transcription. Recent advances point towards the combinatory action of transcription factors, which could account for acrophase or amplitude changes in oscillatory transcripts in a tissue-specific manner. For example, the hepatocyte nuclear factor 4A (HNF4A) interacts with the core clock complex at shared chromatin sites in the mouse liver, suggesting a regulatory mechanism in which HNF4A opposes CLOCK:BMAL1 transactivation at specific metabolic genes [20] (Fig. 1b). This molecular mechanism is also involved in circadian reprogramming in pathological states, as described for the TF NF-κB, which relocates CLOCK:BMAL1 heterodimers to new genomic sites in response to inflammatory signals in the mouse liver [21]. Also, PPARα (peroxisome proliferator-activated receptor alpha) and SREBP (sterol regulatory element-binding protein 1) mediate a major rewiring of the hepatic circadian transcription of lipid-related genes in a mouse model of diet-induced obesity [22].

## Epigenetic control of circadian transcription

Transcriptional cycles governed by the molecular clock occur in the chromatin fiber. It is conceptually apparent that the circadian machinery needs assistance from many chromatin factors to regulate circadian oscillations. Up-and-coming research deciphers some of these interacting factors, and recent technological developments, including high-throughput sequencing and advanced microscopy, are further contributing. Therefore, epigenetic regulation is now considered fundamental for the correct timing in gene expression. Perhaps, the first evidence implicating epigenetic regulation in the control of circadian rhythmicity was the observation of light-induced phosphorylation of histone H3 at Serine 10 (H3S10) in the central clock [23]. Remarkably, high throughput analyses have enlightened a rhythmic epigenome in different tissues. For example, neuronal network properties in the SCN display high plasticity in response to light/dark cycle length. Interestingly, SCN adaptation to distinct light periods relies on DNA methylation patterns at specific genes related to neurotransmitter receptors and ion channels, including several potassium, calcium, and GABA channels [24, 25]. Notably, cytosine methylation oscillates also in peripheral tissues, such as the lung and liver, and epigenetic variation due to methyl-cytosine oscillation is evident during aging [26]. Besides DNA methylation, histone posttranslational modifications (PTMs) have been implicated in cyclic transcription, including histone acetylation or distinct histone methylation patterns, all of which have been shown to oscillate at multiple genomic regions (Fig. 1b). For example, circadian rhythms in histone acetylation were first described in the promoter regions of *Per1*, *Per2*, and *Cry1* genes, and a rhythmic interaction of the histone acetyltransferase (HAT) p300 with CLOCK appeared to be responsible for it [27]. Indeed, the evidence of HAT activity within the CLOCK protein reinforces the notion of an epigenetic component in the circadian clock [28]. So far, circadian rhythms in histone PTMs have been widely described in mouse liver [8, 29–31]. For example, oscillations in transcriptionally activating marks H3K4me3 and H3K9ac are apparent in the promoters of many rhythmic genes. Concomitantly, H3K9me3 repressive mark also displays circadian rhythmicity. ChIP experiments using a combination of antibodies recognizing active and inactive RNAPII, and the elongation marks H3K79me2 and H3K36me3 reveal circadian control exerted on RNAPII recruitment, activation, and elongation steps [8, 29]. Nonetheless, rhythmic loading of RNAPII to promoters rather than its activation from a poised state is mostly responsible for cyclic gene expression in the mouse liver [32].

It is considered that the molecular clock coordinates with specific epigenetic remodelers to sustain the circadian dynamics of the epigenome [33–39]. For example, the histone methyltransferase mixed lineage leukemia 1 (MLL1) interacts with CLOCK:BMAL1 and imposes oscillatory pattern to the H3K4me3 activating epigenetic mark at the promoters of *Dbp* and *Per1* [38]. Interestingly, MLL1 circadian enzymatic activity appears to be directed by posttranslational modifications consisting of rhythmic acetylation at K1130 and K1133 [39]. MLL1 acetylation potentiates its catalytic activity, while deacetylation mediated by the deacetylase sirtuin1 (SIRT1) is inhibitory; thereby, cyclic MLL1 acetylation can be detected in mouse embryonic fibroblasts and mouse liver [39]. Similarly, oscillatory histone acetylation is assisted through partnering with the HATs p300 and CBP, which appear rhythmically recruited to specific genomic loci [8, 27, 40, 41]. Additional chromatin remodelers involved in circadian control include the NuRD (nucleosome remodeling deacetylase) transcriptional corepressor complex, which contains the ATP-dependent nucleosome remolding CHD4 (chromodomain-helicase-DNA-binding protein 4) [42]. Hence, NuRD corepressor interacts and assists PER complexes to silence transcription [42]. A rhythmic epigenome is central to determine the cell-type-specific circadian transcriptional output, ultimately imposing oscillations on biological pathways that temporally segregate metabolism around the day. For example, the histone deacetylase HDAC3 synchronizes circadian lipid and glucose metabolism in mouse liver and muscle and is rhythmically recruited to chromatin within a repressor complex directed by the circadian protein REV-ERBα [43–45]. Rhythmic HDAC3 recruitment directs oscillations in H3K9 acetylation and chromatin compaction at lipid biosynthetic genes, a molecular mechanism underlying the diurnal rhythm observed in hepatic lipogenesis [45].

Environmental cues altering circadian rhythms prompt a major reprogramming of the circadian transcriptome where epigenetic mechanisms become essential [46]. It is conceptually evident that the clock machinery senses metabolic states to adapt its function accordingly, and several epigenetic mechanisms have been described linking metabolic cues with the molecular clock [6]. For example, SIRT1 deacetylase activity is coupled to the hydrolysis of the metabolite NAD^+^ (nicotinamide adenine dinucleotide, oxidized) in a way that endogenous fluctuations of NAD^+^ levels determine SIRT1 function [47]. Because SIRT1 also regulates clock function, it is considered a nutrient sensor that connects intracellular energy state to the clock [46]. Interestingly, JmJC domain-containing histone demethylases such as KDM5A (lysine-specific demethylase 5A) relate oxygen sensing to chromatin in a way that, in hypoxic conditions, H3K4me3 and H3K36me3 activating histone modifications are increased [48, 49]. Indeed, KDM5A participates in circadian oscillator function and influences histone acetylation levels at the *Per2* promoter [50]. It is tempting to speculate that KDM5A functions as an oxygen sensor connecting epigenetic responses to the circadian clock in normoxic and hypoxic conditions [51, 52].

Intriguingly, latest discoveries pinpoint an unexpected epigenetic regulatory layer of circadian transcription, being higher order genome topology and nuclear architecture (Fig. 1b). Not only do epigenetic mechanisms regulate cyclic transcription, but they also establish chromatin loops or long-range interactions and determine the spatial distribution of circadian genes in the nuclear space. To introduce the current understanding on nuclear architecture, we have discussed it in the next section. Thereafter, we have dissected the regulatory role of chromatin topology in circadian control of gene expression.

## Spatial organization of the genome

How the genome organizes inside of the nucleus has been a permanent question for the chromatin biology field. The nucleosome is considered as the basic unit of the chromatin and is composed by a histone octamer wrapped by 145 to 147 base pairs of DNA [53]. However, a human diploid cell nucleus contains roughly 30 million nucleosomes, which must be somehow organized to provide the framework for the genome functions. Basically, the combination of two technical approaches has provided the knowledge on these major questions; these are microscopy approaches and chromosome conformation capture (3C)-based techniques. Remarkably, outstanding and recent advances in photonics, biophysics, next-generation sequencing, single cell analyses, and computational approaches have set the basis for our current and profound understanding of genome folding within the nuclear space [54]. Still, many questions remain to be addressed, amongst these are the precise impact of nuclear architecture on genome functions such as transcription or DNA repair, or the dynamics of the 3D genome during processes occurring at different time scales, such as cell cycle, cellular differentiation, environmental adaptation, or circadian cycles.

**Fig. 2:**
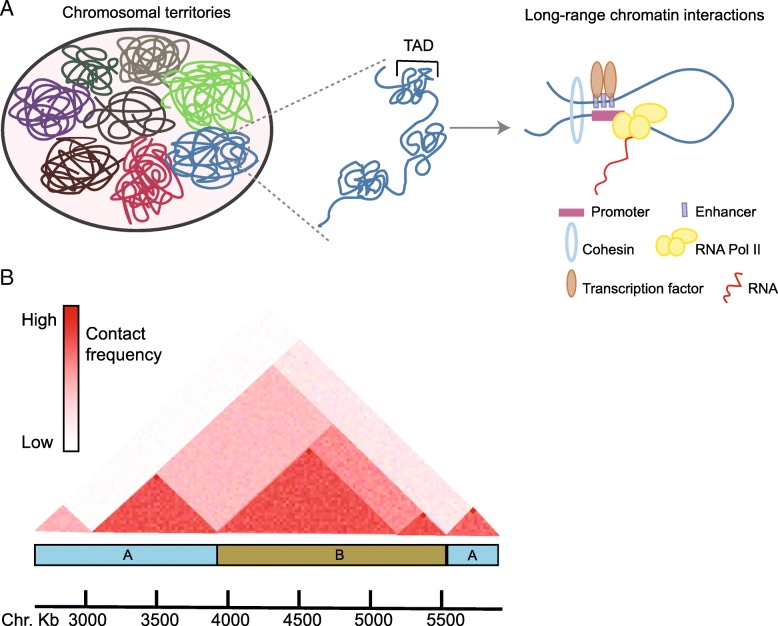
Levels of chromatin organization. **a** Interphasic chromosomes occupy discrete areas inside of the nucleus, termed chromosomal territories. At the sub-chromosome level, chromatin is organized in topologically associating domains (TADs), considered as fundamental building blocks of the three-dimensional genome. TADs are remarkably conserved between cell types and even species and span hundreds of kilobases to several megabases. Notably, long-range chromatin interaction, such as enhancer-promoter contacts, does not cross TAD boundaries, meaning that intra-TAD interactions are frequent, while inter-TAD contacts are unusual. Specific architectural proteins consolidate three-dimensional contacts, including transcription factor binding, Cohesin, CTCF, Mediator, or YY1 (not shown). **b** Representation of a contact heat map generated from a HiC experiment. TADs visually appear as triangles limiting a portion of the genome where interactions are very frequent (red color). Each TAD pertains to either A (blue) or B (green) compartment, which are estimated by an eigenvector analysis of the genome contact matrix after normalization of the HiC data. A compartment correlates with open, transcriptionally active chromatin, while B compartment presents repressive features

Direct evidences of the arrangement of chromosomes in interphase were provided by the visualization of chromosome territories (CT) using fluorescence in-situ hybridization techniques (FISH) [55] (Fig. 2a). Particularly, labeling chromosomes simultaneously in single cells using a combination of fluorochromes reveals that each chromosome occupies a specific area within the nucleus [56]. Interestingly, chromosome positioning is nonrandom and displays evolutionary conserved features [57]. For example, gene-rich and small-sized chromosomes are located closer to the center of the nucleus than gene-poor or larger chromosomes, which are generally displaced towards the periphery [58–60]. The organization of chromosomes in discrete areas is further confirmed by the 3-C derivate techniques, 4C and Hi-C, where contacts within chromosome are much more frequent than between different chromosomes [61–63]. Indeed, functional relevance to chromosome positioning might be related to transcriptional rates, as actively transcribed genes are preferentially in a more internal position [64].

Principles of genome folding have been uncovered using Hi-C techniques, and the organization into two main structural and functional compartments, termed A and B, is apparent. Technically, A/B compartments are determined by a principal component analysis of normalized Hi-C data contact matrices [63, 65] (Fig. 2b). Structurally, each compartment behaves as a unit containing either open, transcriptionally active chromatin (A compartment) or silent, heterochromatic regions (B compartment). Moreover, chromatin within A compartment is gene dense and enriched in activating epigenetic marks, while compartments of type B segregate regions much more resistant to DNAse digestion and marked with heterochromatic histone modifications, such as H3K9me3. Functionally, A and B compartments correlate with early and late replications times respectively and segregate chromatin regions with distinct transcriptional states [66]. At the megabase and sub-megabase scales, the genome appears organized in segments with very high interaction frequencies within, separated from each other by boundaries which sharply limit the interactions between them. These structural units are termed topologically associating domains (TADs), which spatially divide the linear genome into three-dimensional units (Fig. 2). Inside each TAD, numerous chromatin loops arrange the specific enhancer-promoter interactions, positioning of insulator elements, functionality of locus control regions, etc. Interestingly, the boundaries limiting TADs in mammals are largely constant across different cell types [67–69]. These boundaries provide isolation to regulatory elements such as enhancers, in a way that they influence expression of specific genes within the same TAD, but are excluded from contacting and regulating genes pertaining to any other TAD [70–72]. The architectural proteins CTCF (CCCTC-binding factor) and cohesin play major roles in maintaining genome topology, but other factors such as the Mediator complex, YY1 (Ying Yang 1), and even non-coding RNAs also intervene, while a constant source of ATP is necessary for adequate establishment of loop domains [73–76] (Fig. 2).

Recent research point towards a crucial role for transcription factors (TFs) in shaping genome topology during cell reprogramming and differentiation. For example, cell-type-specific contacts between loci bound by distinct transcription factors are apparent during neural differentiation [77]. Some of these TFs include the neural master regulator Pax6 (Paired box 6) or the neuronal TFs NeuroD2 and TBR1 (T-box, brain, 1) [77]. Moreover, the TF Pax5 (Paired box 5) drives lineage-specific genome architecture during B cell differentiation [78]. Remarkably, the Yamanaka factors OCT4 (octamer-binding transcription factor 4), NANOG (Nanog homeobox), and SOX2 (sex determining region Y-box 2) dictate gene regulation during B cell reprogramming by inducing sequential changes in genome topology and chromatin states [79]. Indeed, chromatin accessibility measured by ATAC-seq and H3K4me2 dynamics is initially lost at lineage-specific gene-regulatory elements, and subsequently an open chromatin state is established at pluripotency genes [79]. This reprogramming trajectory is accompanied by variations in genome compartmentalization which are coherent with the observed transcriptional rewiring [79]. Collectively, these evidences implicate TFs as drivers of topological fluctuations in the genome, and genome topology as a framework for delineating cell fate in mammals. However, how transcriptional reprogramming relates to nuclear architecture during cellular processes requiring shorter time scales remains highly elusive. In this line, the circadian program of gene expression provides the ideal scenario to uncover these questions.

## Beyond circadian transcription: genome topology assists rhythmicity

It is being increasingly recognized that the spatial organization of the genome in the interphase nucleus constitutes a regulatory layer coordinating chromatin functions, and the circadian program of gene expression is not an exception. Increasing evidence demonstrates that spatial organization of circadian genes in the nucleus assists rhythmic expression [6].

Perhaps the first evidence suggesting circadian rhythms in DNA topology comes from studies in the green alga *Chlamydomonas reinhardtii* [80], where the DNA supercoiling in the chloroplast was found to oscillate with a diurnal rhythm. Moreover, this endogenous fluctuation tightly correlates with circadian gene expression in the chloroplast, suggesting a direct link between DNA topology and rhythmic transcription [80]. A similar scenario has been described in the unicellular cyanobacterium *Synechococcus elongatus*, where the circadian clock controls transcription of virtually the entire genome [81]. In general, the bacterial chromosome is compacted into a highly organized structure termed “nucleoid,” based on condensation and coiling of DNA [82]. Interestingly, *S*. *elongatus* shows circadian rhythms in chromosome compaction which can drive oscillations in gene expression and determine the circadian transcriptional output [83–85]. These topological rhythms in the nucleoid are directed by the endogenous clock [84, 85].

In *Arabidopsis thaliana*, the master zeitgeber, light, can trigger rapid spatial repositioning of a group of circadian genes including *CAB* (chlorophyll a/b-binding protein), *RBCS* (ribulose-1,5-bisphosphate carboxylase/oxygenase small subunit), and *PC* (phytochelatin synthase), relocating them from the nuclear interior to the periphery, thus leading to transcription [86]. Along the same line, exposure to light during germination triggers massive changes in nuclear architecture and RNA PolII relocation, a process involving the circadian cryptochrome photoreceptors [87, 88]. These evidences indicate that the spatial organization of the genome inside of the nucleus is influenced by the circadian clock. Interestingly, the spatial positioning of the core clock gene *Bmal1* in mammals might be important for circadian control, as the promoter of this gene is bound by the nuclear membrane protein MAN1 (MAN antigen 1), thereby activating its transcription [89]. Additionally, genetic disruption of certain components of the nuclear envelope such as LMN1B (lamin 1-B) or LBR (lamin B receptor) impact circadian amplitude and period length [89], strengthening a role for nuclear architecture in assisting the circadian oscillator. Indeed, in human HCT116 cells, the cyclic gene PARD3 (partitioning defective 3 homolog) is rhythmically recruited to the nuclear periphery through a molecular interplay driven by CTCF and PARP1 (poly [ADP-ribose] polymerase 1) [90].

**Fig. 3:**
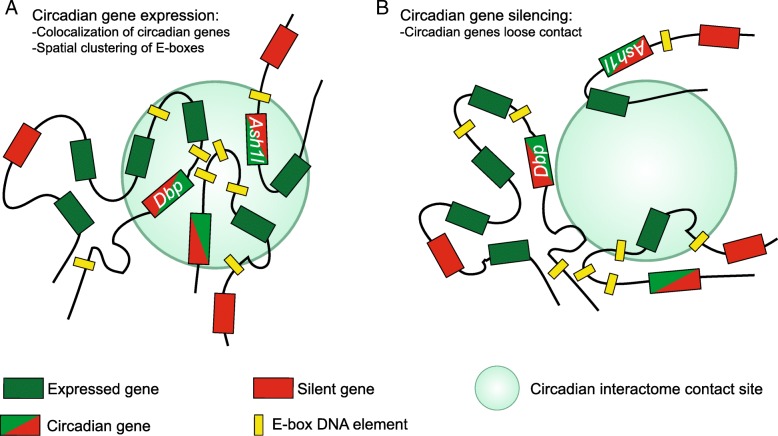
A circadian interactome assists rhythmic gene expression. **a** Cyclic expression of circadian genes such as *Dbp* and *Ash1l* is physically coupled in a nuclear environment enriched for actively transcribed genes and E-box DNA elements, coincident with the presence of clock-specific transcription factories. **b** When clock-controlled genes become silent during the circadian cycle, their physical distances in the nucleus increase, hence losing contact. The circadian interactome is directed by the clock machinery and depends on a functional BMAL1 [61]

Using chromosome conformation capture on ChIP (4C) technology, it was demonstrated that circadian genes in mouse embryonic fibroblasts (MEFs) are organized in functional territories inside of the nucleus (Fig. 3) [61]. Interestingly, the circadian clock has a role in fine tuning certain temporal changes in genome organization. Using the circadian gene *Dbp* as a bait for 4C analyses, it was shown that cyclic chromosomal arrangements accompany circadian transcription, hence delineating a circadian interactome [61]. These genomic interactions are much less dynamic in MEFs (mouse embryonic fibroblasts) lacking the core clock gene *Bmal1*, indicating a prevalent role for the circadian machinery in supporting fluctuations in the nuclear landscape. Some of the genes located at the *Dbp* circadian interactome are rhythmically transcribed, and their phase closely follows that one from *Dbp*. These evidences suggest that CLOCK:BMAL1-directed transcription occurs in dedicated transcription factories. Supporting this idea, a significant spatial congregation of circadian E-box elements is observed around *Dbp* gene [61]. Hence, as previously reported for different transcriptional networks, the circadian program of gene expression in MEFs happens in pre-established nuclear domains (Fig. 3) [6, 91]. Additional transcription factor binding sites are highly represented in the *Dbp* circadian interactome, indicating that combinatorial associations with multiple DNA elements provides a spatial framework for circadian transcription. Intriguingly, the genes in physical proximity from *Dbp* pertain to biological pathways known to be circadian, such as chromatin regulation or xenobiotic detoxification, suggesting the presence of specialized transcription factories regulating circadian gene networks.

These evidences have been reinforced using a whole genome approach termed HiC in human fibroblasts, where temporal dynamics in nuclear architecture is also apparent [92, 93]. Indeed, relative physical distances between the core clock genes *PER2* and *CLOCK* during the circadian cycle serve as a predictor of their transcriptional activity [92]. Moreover, several circadian interactomes with co-regulated circadian genes also appear in human fibroblasts, suggesting that specialized and dynamic nuclear hubs participate in the regulation of circadian transcription [92]. Intriguingly, in both human and mouse-derived fibroblasts, the H3K36 methyltransferase coding gene *ASH1L* (*Ash1l*; absent, small, or homeotic-like) is dynamically coupled inside a circadian interactome with additional rhythmically expressed and cell-type-specific genes (Fig. 2), strongly suggesting a role for ASH1L in chromatin remodeling associated to circadian nuclear topology [61, 92].

## Mammalian circadian genome topology: lessons from mouse liver

**Fig. 4:**
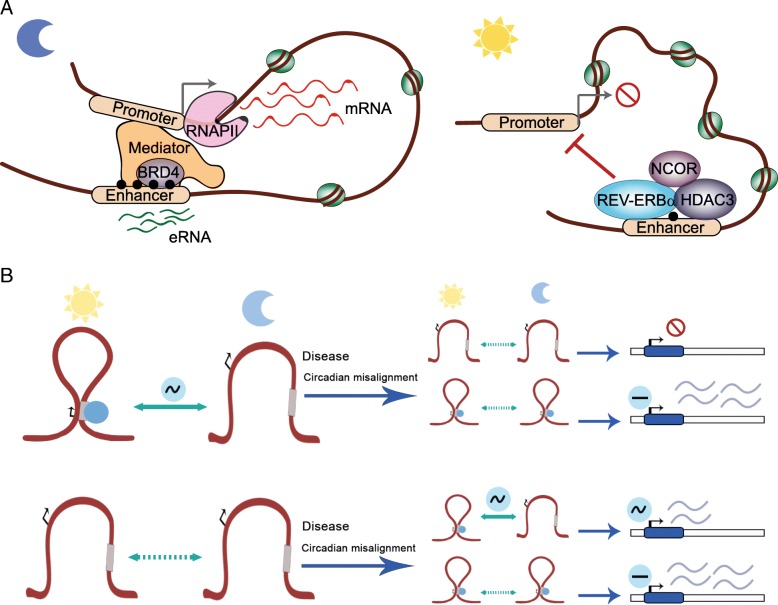
Circadian chromatin interactions in health and disease. **a** Many circadian long-range interactions direct enhancer-promoter contacts at a specific time of day and in a tissue-specific manner. For example, in the mouse liver, *Cry1* promoter contacts an enhancer element located within an intron, specifically at ZT22 (night time). This interaction is characterized by H3K27ac mark at the enhancer (back dots), which is recognized by BRD4 and recruits the Mediator complex. eRNAs also appear at ZT22, and *Cry1* gene is therefore transcribed. During the opposite phase, at ZT10 (day time), the clock protein REVERBα displaces Mediator and BRD4 from the enhancer region and recruits a repressor complex containing NCOR and the deacetylase HDAC3. The active enhancer marks H3K27ac and eRNA decrease and *Cry1* transcription ceases [95]. **b** During the day, some chromatin interactions oscillate while others remain constant. Misalignment of circadian rhythms or certain pathological states could trigger different scenarios: circadian contacts could be disrupted leading, for example, to a permanent interaction of the absence of it (upper panel). Additionally, some new interactions may appear, either oscillatory or not (lower panel). These alterations may cause distinct patterns of gene expression as observed in certain pathological conditions, including cancer and cognitive or metabolic diseases

Recent studies in mouse liver demonstrate that dynamic genome topology shapes circadian gene transcription. Indeed, HiC and 4C techniques reveal that circadian chromatin loops happen in cis to coordinate liver-specific enhancer-promoter interactions, driving transcription [9, 94, 95]. This has been demonstrated for the clock gene *Cry1*, which depicts a dynamic loop between the TSS and an enhancer located within the first *Cry1* intron [94, 95]. This contact appears at night (ZT20-22; ZT stands for zeitgeber time), preceding the peak of expression of *Cry1*. Indeed, genetic ablation of the intronic region involved in this dynamic contact in mice impacts circadian behavior by shortening period length, because of constitutive *Cry1* gene silencing [94] (Fig. 4a). Remarkably, this circadian interaction is driven by the clock molecular machinery, and particularly, by rhythmic REV-ERBα binding during the daytime, which in turn impedes loop formation. Interestingly, REV-ERBα functionally opposes enhancer-promoter loop formation at a number of rhythmic genes in the liver by obstructing recruitment of the general looping factor mediator (MED1) and the reader of H3K27ac BRD4 (Bromodomain-containing protein 4) (Fig. 4a) [95]. Importantly, most of BMAL1-target genes transcriptional oscillations in the mouse liver rely on functional interactions between regulatory elements, which appear increased during the phase of BMAL1 recruitment to chromatin, around ZT6 [96]. Together, these evidences highlight the fundamental role of chromatin looping in regulating circadian transcription and identify new molecular actors which are critical to maintain dynamic chromosomal interactions.

A large amount of genomic and epigenomic data in the circadian field has been generated from mouse liver, while information from other tissues is mostly lacking. However, the circadian transcriptome has been explored across many tissues. A comparative analysis that integrates TF binding site predictions with rhythmic mRNA accumulation suggests that the clock TFs can colocalize with tissue-specific TFs, such as FOXA2 (forkhead box protein A2) and HNF6 (hepatocyte nuclear factor 6) in the liver, which in turn can mediate tissue-specific looping, thereby shaping a particular circadian transcriptome for each tissue or cell type [9, 96]. This notion is further reinforced by the identification of circadian activity of enhancer regions based on enhancer RNA (eRNA) transcription [97, 98]. It is generally accepted that these non-coding RNAs are implicated in long-range looping interactions and constitute a molecular signature of active enhancers [98]. Indeed, eRNAs expressed between ZT6-ZT9 are enriched in E-boxes, while those accumulated around ZT18-ZT24 contain RevDR2 and RORE motifs. Interestingly, circadian eRNAs in the mouse liver are constitutively enriched for the Forkhead box (FOX) and HNF4 motifs [97]. Since eRNAs are most probably located at looping sites, it is tempting to speculate that FOXO1 (forkhead box protein O1) and HNF4 TFs could also mediate liver-specific chromatin interactions.

An important question remains whether enhancer-promoter interactions could be the trigger of distinct genetic programs of gene expression in response to environmental challenges [46]. Recent research points into this direction. For example, in the mouse liver, feeding-mediated gene repression is accompanied by decreased H3K27ac mark at sites enriched for motifs known to interact with the glucocorticoid receptor (GR), cAMP responsive element binding protein (CREB), and FOX TFs [99], suggesting that these could mediate transcriptional reprogramming in response to feeding through dynamic chromatin looping. Moreover, circadian eRNAs are reprogrammed upon high-fat diet feeding, and some new eRNAs become oscillatory, while others display phase shifts. Interestingly, SREBP1 and PPARα TFs are required for cyclic expression of newly oscillating eRNAs in high-fat diet fed mice near some de novo lipogenesis and fatty acid oxidation genes, such as *Fasn* (fatty acid synthase), *Acaca* (acetyl-CoA carboxylase 1), *Acox1* (acyl-CoA oxidase 1), or *Aldh3a2* (aldehyde dehydrogenase 3 family member A2) [22]. In this scenario, it is tempting to speculate that alterations in genome topology or the arrangement of certain contact patterns between critical regulatory elements may establish a specific chromatin conformation leading to the circadian transcriptional reorganization observed under many pathological conditions, including metabolic diseases, cognitive disorders, or even cancer (Fig. 4b) [100–102].

## Conclusion

The dynamic interplay between spatial organization of the genome and the circadian gene expression program is emerging. Up-and-coming research highlights a remarkable plasticity on chromatin states and genome dynamics during the circadian cycle, and epigenetic transitions appear to be at the core of transcriptional reprogramming triggered by environmental cues. Components of the molecular clock, including BMAL1 and REVERBα, emerge as important regulators of dynamic interactions in the three-dimensional genome during the circadian cycle. However, the role of the circadian machinery in defining functional regulatory elements in response to environmental challenges remains to be understood. Growing our understanding on these mechanisms will remarkably benefit from recent technological advances on molecular imaging of live cells [103]. For example, imagining endogenous loci using fluorescently labeled CRISPR/dCas system in live cells could provide means to reveal the significance of spatiotemporal organization and dynamics of chromatin during the circadian cycle. Moreover, intracellular distribution of metabolites serving as coenzymes for epigenetic regulators, such as NAD^+^, is generally diminished [104]. Hence, it will be of interest to determine if local concentrations of specific metabolites in nuclear microenvironments control the activity of epigenetic remodelers in a highly dynamic and locally restricted regulatory level.

All these findings provide an exciting scenario to investigate circadian regulation of pathological states and constitute starting points to uncover precise molecular links that couple the circadian clock with metabolic control, epigenetic regulation, and environmentally triggered phenotypes (Fig. 4b). For example, further research is necessary to reveal functional relationships between the molecular clock and the epigenetic reprogramming associated to distinct feeding patterns, which could pave the way to highly effective chronotherapeutic approaches for the treatment of metabolic pathologies. Finally, advancing our knowledge along these lines could provide means to further understand the implications of genetic variation within non-coding regulatory elements for human disease [105].

## Abbreviations

3C: Chromosome conformation capture
Acaca: Acetyl-CoA carboxylase 1
Acox1: Acyl-CoA oxidase 1
Aldh3a2: Aldehyde dehydrogenase 3 family member A2
ASH1L: (Absent, small, or homeotic)-like
ATAC-seq: Assay for transposase-accessible chromatin sequencing
ATP: Adenosine triphosphate
BMAL1: Brain and muscle ARNT-like protein-1
BRD4: Romodomain-containing protein 4
CAB: Chlorophyll a/b-binding protein
CHD4: Chromodomain-helicase-DNA-binding protein 4
ChIP: Chromatin immunoprecipitation
CLOCK: Circadian Locomotor Output Cycles Kaput
CREB: cAMP responsive element binding protein
CRY: Cryptochrome
CT: Chromosome territories
CTCF: CCCTC-binding factor
DBP: Albumin-D-site-binding protein
DNA: Deoxyribonucleic acid
eRNA: Enhancer RNA
Fasn: Fatty acid synthase
FISH: Fluorescence in-situ hybridization
FOX: Forkhead box
FOXA2: Forkhead box protein A2
FOXO1: Forkhead box protein O1
GR: Glucocorticoid receptor
H3: Histone 3
HAT: Histone acetyltransferase
HDAC3: Histone deacetylase 3
HLF: Hepatic leukemia factor
HNF4A: Hepatocyte nuclear factor 4A
HNF6: Hepatocyte nuclear factor 6
KDM5A: Lysine-specific demethylase 5A
LBR: Lamin B receptor
LMN1B: Lamin 1-B
MAN1: MAN antigen 1
MEF: Mouse embryonic fibroblasts
MLL1: Mixed lineage leukemia 1
NAD^+^: Nicotinamide adenine dinucleotide, oxidized
NANOG: Nanog homeobox
NFIL3: Nuclear factor, interleukin-3 regulated
NF-κB: Nuclear factor kappa-light-chain-enhancer of activated B cells
NR: Nuclear receptor
NuRD: Nucleosome remodeling deacetylase
OCT4: Octamer-binding transcription factor 4
PAR-bZip: Proline and acidic amino acid-rich basic leucine zipper
PARD3: Partitioning defective 3 homolog
PARP1: Poly [ADP-ribose] polymerase 1
Pax5: Paired box 5
Pax6: Paired box 6
PC: Phytochelatin synthase
PER: Period
PPARα: Peroxisome proliferator-activated receptor alpha
PTM: Posttranslational modifications
RBCS: Ribulose-1,5-bisphosphate carboxylase/oxygenase small subunit
REV-ERB: Reverse erythroblastosis virus
RNA: Ribonucleic acid
RNAPII: RNA polymerase II
ROR: Retinoic acid-related orphan receptor
RORE: Retinoic acid-related orphan receptor-binding element
SCN: Suprachiasmatic nucleus
SIRT1: Sirtuin1
SOX2: (Sex determining region Y)-box 2
SREBP1: Sterol regulatory element-binding protein 1
TAD: Topologically associating domain
TBR1: T-box, brain, 1
TEF: Thyrotroph embryonic factor
TF: Transcription factor
TSS: Transcription start site
TTFL: Transcriptional/translational feedback loops
YY1: Yin Yang 1
ZT: Zeitgeber time
